# Coastal polynyas: Winter oases for subadult southern elephant seals in East Antarctica

**DOI:** 10.1038/s41598-018-21388-9

**Published:** 2018-02-16

**Authors:** Sara Labrousse, Guy Williams, Takeshi Tamura, Sophie Bestley, Jean-Baptiste Sallée, Alexander D. Fraser, Michael Sumner, Fabien Roquet, Karine Heerah, Baptiste Picard, Christophe Guinet, Robert Harcourt, Clive McMahon, Mark A. Hindell, Jean-Benoit Charrassin

**Affiliations:** 1 Sorbonne Universités, UPMC Univ., Paris 06, UMR 7159 CNRS-IRD-MNHN, LOCEAN-IPSL, 75005 Paris, France; 2 Institute for Marine and Antarctic Studies, University of Tasmania, Private Bag 129, Hobart, Tasmania 7001 Australia; 3 Antarctic Climate & Ecosystems Cooperative Research Centre, University of Tasmania, Private Bag 80, Hobart, Tasmania 7001 Australia; 4 National Institute of Polar Research, 1-9-10, Kaga Itabashi-ku, Tokyo, 173-8515 Japan; 5 SOKENDAI (The Graduate University for Advanced Studies), Tachikawa, Tokyo, 190-8518 Japan; 6 Australian Antarctic Division, Channel Highway, Kingston, Tasmania 7050 Australia; 7 British Antarctic Survey, High Cross, Cambridge, CB3 0ET UK; 8 Institute of Low Temperature Science, Hokkaido University, N19 W8, Kita-ku, Sapporo, 060-0819 Japan; 9 Department of Meteorology, Stockholm University, S-106 91 Stockholm, Sweden; 10 Centre d’Etudes Biologiques de Chizé (CEBC), UMR 7372 Université de la Rochelle-CNRS, 79360 Villiers en Bois, France; 11 Department of Biological Sciences, Macquarie University, Sydney, New South Wales 2109 Australia; 12 Sydney Institute of Marine Science, 19 Chowder Bay Road, Mosman, New South Wales 2088 Australia

## Abstract

Antarctic coastal polynyas are regions of persistent open water and are thought to be key bio-physical features within the sea-ice zone. However, their use by the upper trophic levels of ecosystems remains unclear. A unique bio-physical dataset recorded by southern elephant seals reveals that East Antarctic polynyas are a key winter foraging habitat for male seals. During their post-moult trips from Isles Kerguelen to the Antarctic continental shelf, a total of 18 out of 23 seals visited 9 different polynyas, spending on average 25 ± 20% (up to 75%) of their total trip time inside polynyas. Changes in seal foraging and diving behaviours are observed inside polynyas as compared to outside polynyas. Two polynya usages by seals are observed for the inactive and active polynya phases, pointing to different seasonal peaks in prey abundance. During the active polynya phase, we link seal foraging behaviour to changes in the physical stability of the water-column, which likely impact the seasonal biological dynamics within polynyas.

## Introduction

Coastal polynyas are persistent and recurrent regions of open water (i.e. no ice, thin ice, or reduced ice concentration) that occur within the polar sea-ice zone, ranging in size from tens to tens of thousands of square kilometers^1^. Antarctic coastal polynyas are largely latent heat polynyas because they are mechanically driven and form in regions of divergent ice motion due to prevailing winds, oceanic currents, and/or dynamical barriers (e.g., headlands, floating glaciers, fast-ice, grounded icebergs) blocking the passage of pack ice. Such conditions promote the loss of heat from the ocean to the atmosphere, and the formation of new sea-ice^2,3^. Surface waters associated with polynyas often have relatively high biological production as they are the first polar marine systems in spring to be exposed to solar radiation; either because they are not covered by sea-ice or their thin ice is more susceptible to early breakout^4^. The associated phytoplankton blooms, which persist in summer after sea-ice has melted away, maintain the highest phytoplankton biomass on the continental shelf^4^. The edges of polynyas, characterized by complex and deformed sea-ice, provide favourable habitat for sea-ice algae grazers (refs^5–8^; reviewed by ref.^9^). While enhanced primary production occurs primarily between early spring and summer, in polynyas, zooplankton growth and reproduction is extended into late summer and early autumn^9^. Enhanced vertical carbon flux in polynyas can also support rich benthic communities^10,11^. Consequently, Antarctic coastal polynyas provide favourable physical and biological conditions for productive marine ecosystems accessible to air-breathing populations of seabirds and marine mammals to feed in throughout the ice season^4,12–14^.

Previous studies have investigated the timing of primary production within Antarctic polynyas and the controlling environmental factors (*e*.*g*.^4,13^, and^14^). However, the seasonal usage of polynyas by mid- to upper trophic levels is still poorly known (except for the massive Ross Sea polynya which is well documented and studied, *e*.*g*.^12^), as are the underlying biophysical mechanisms that make these regions profitable for foraging. It is unclear if and how marine mammals and seabirds use polynyas either as an air-breathing refuge or as foraging habitat during winter. The role of polynyas as a predictable open water access to food has been suggested (*e*.*g*. Emperor penguins;^15–17^; Adélie penguins^4,18^); acting to reduce commute time and energy expenditure between colonies and food supply. The spring/summer use of polynyas by upper trophic levels^19^ can be associated with the seasonal primary production bloom, for example, the magnitude of primary production in polynyas was positively linked with Adélie penguin colony size^4^ and the probability of Weddell seals producing pups^20^. However, the ecological role of each polynya differs between regions (e.g.^13,21^), and might be associated with local environmental conditions, such as bathymetry, ‘icescape’ (configuration of coastline topography, ice shelves/glaciers, fast ice and grounded icebergs), hydrological properties, polynya size and sea-ice production, some of which vary seasonally. Understanding the role of polynyas in the ecosystem requires a thorough bio-physical assessment of the polynya environment throughout the year, which is logistically challenging during the active sea-ice growth period (March-October). Although a polynya effectively disappears during spring-summer when the sea ice melts around it, its impact on the water column remains and we therefore also pay attention to this period, referred to hereinafter as the “pre/post-polynya” phase.

We investigated the use of coastal polynyas in East Antarctica by a common mesopelagic predator of the Southern Ocean, the southern elephant seal (*Mirounga leonina*; SES). Circumpolar deep-diving animals, SES come ashore twice-yearly to breed and moult^22^. Otherwise, SES spend 80% of their time at-sea in transit between their colonies on sub-Antarctic Islands and their foraging grounds^23,24^. Key foraging habitat for SES have been found to be sex-dependent^25,26^, as well as variable amongst breeding subpopulations^27,28^. Among SES from the Kerguelen Islands, there are two main post-moult (i.e. winter) foraging strategies: some individuals use the Kerguelen shelf and/or frontal systems of the Antarctic Circumpolar Current (ACC), while others travel south to sea-ice covered areas and the Antarctic shelf^25,26^.

Despite not being considered an “ice obligate” species, some SES spend their entire post-moult foraging trip (January-October) within the Antarctic sea-ice region^26,28–31^. In particular, male SES can remain over the Antarctic shelf despite dense and persistent pack and fast ice, and it has been hypothesized they might use open water areas between the pack and the fast ice^19,32,33^. Within coastal polynyas, they are able to take advantage of both the enriched ecosystems and permanent breathing access during the winter season. Here, we analysed subadult male SES at-sea movements and diving behaviour collected over eleven years (seven years of tracking from 2004 to 2014) to assess (i) whether polynyas were used by SES and if so, the specific timing, (ii) diving behaviour inside polynyas to examine whether they are more favourable foraging grounds than the surrounding shelf/slope areas; and, (iii) the seasonal oceanographic conditions that make these habitats more suitable than adjacent waters.

## Results

### Polynya regional occupation and habitat use

**Figure 1:**
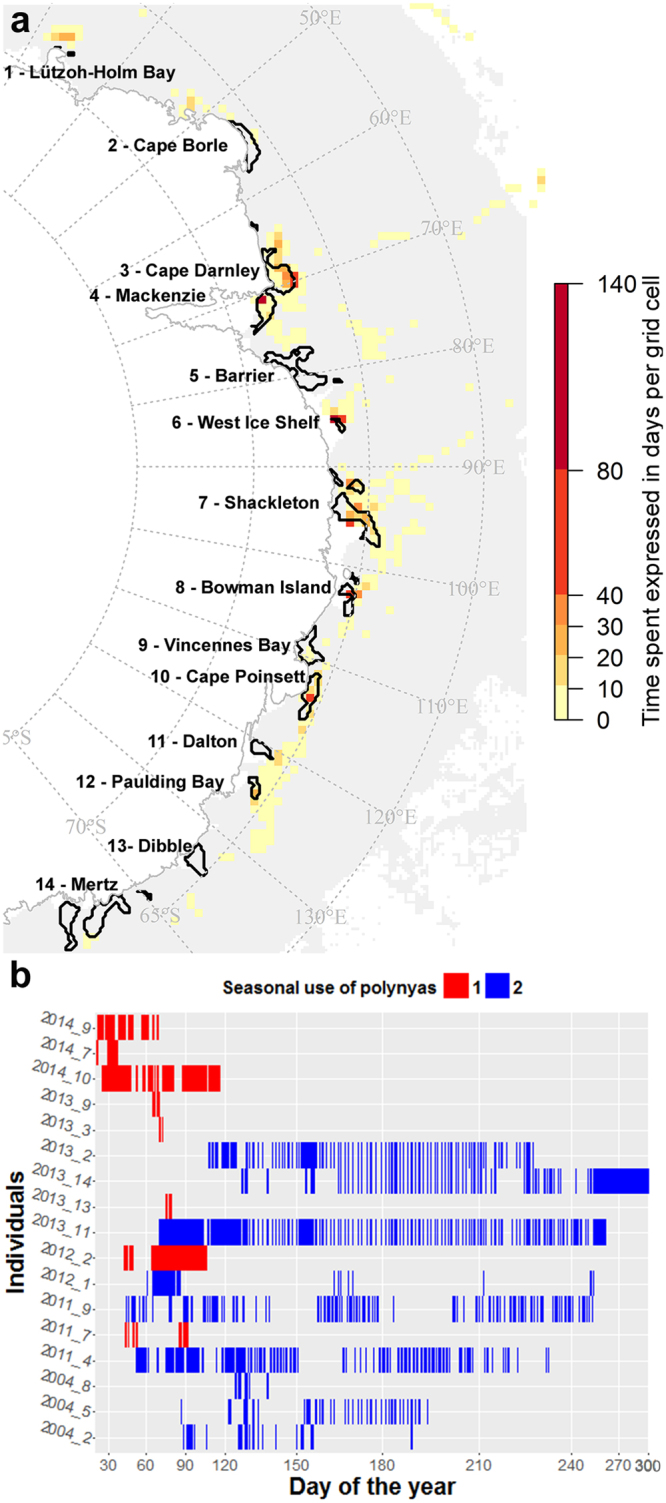
Polynya usage by 17 post-moult Kerguelen male SES from 2004 to 2014 based upon the position of CTD casts (n = 1962). A total of 23 SES (6738 CTD casts) were studied and 18 individuals visited polynyas however CTD data were not available for one individual (2008_1). Panel (a) represents a map of average time spent (days) across all individual seals per grid cell (37.5 × 37.5 km) (expressed in days) computed using the timestamp of CTD casts from 2004 to 2014. Panel (b) represents the daily presence of each seal inside polynyas. The colour scale represents the two seasonal uses of the polynyas among seals with implications for both pre-polynyas (group 1; January–April) and polynyas (group 2; February–October). The map in panel (a) was made by S. Labrousse using R software, version 3.2.4 revised (R Core Team (2016). R: A language and environment for statistical computing. R Foundation for Statistical Computing, Vienna, Austria. https://www.R-project.org/).

A total of 14 polynyas were identified and named following refs^4^ and^17^: 1. Lützoh-Holm Bay, 2. Cape Borle, 3. Cape Darnley, 4. Mackenzie, 5. Barrier, 6. West Ice Shelf, 7. Shackleton, 8. Bowman Island, 9. Vincennes Bay, 10. Cape Poinsett, 11. Dalton, 12. Paulding Bay, 13. Dibble, 14. Mertz. Eighteen of the 23 tracked seals visited 9 of the 14 identified polynya areas from January to November (Fig. 1). Among these 9 polynyas, 4 are “true” coastal polynyas (Mackenzie, West Ice Shelf, Shackleton, Vincennes Bay) while 5 are hybrid that overlap the shelf and the slope regions (Cape Darnley, Bowman Island, Cape Poinsett, Paulding Bay, Mertz). Seal behaviour and polynya use varied between individuals (Table S1): seals spent between 4 to 75% of their total (transmitted) trip inside polynyas, comprising up to 86% of their total time spent over the Antarctic slope/shelf region. Residency within polynyas was primarily located over the shelf (92 ± 16% (mean ± standard error of the mean (SEM)) of time versus 8 ± 16% over the slope, Table S1). When seals were over the shelf region, they spent 46 ± 27% (SEM) of their time inside polynyas while when they were over the slope region they only spent 5 ± 8% of their time inside polynyas (Table S1). When inside polynyas, seals spent 47 ± 26% (SEM) of their time in areas of thin ice (0 to 0.2 m) and the rest in open waters. Based on sea ice production (SIP; see Methods section), seals inside polynyas spent on average 60 ± 29% (SEM) of their time in the core 1 area (2.5 m.y^−1^ ≤ SIP_year_ < 5 m.y^−1^), 32 ± 25% in core 2 (5 m.y^−1^ ≤ SIP_year_ < 10 m.y^−1^) and 8 ± 14% in core 3 (10 m.y^−1^ ≤ SIP_year_). Seals both dived benthically and pelagically (definition of benthic and pelagic dives followed the method developed in ref.^26^) whilst in polynyas (54 ± 34% and 46 ± 34% (SEM) of their time, respectively, Table S1). Inside polynyas, some individuals did show exclusive preference *i*.*e*. 100% pelagic (n = 3) or benthic (n = 1) dives. The different metrics describing the use of polynyas by each seal are given in Table S1.

### Seasonality in polynya use

**Figure 2:**
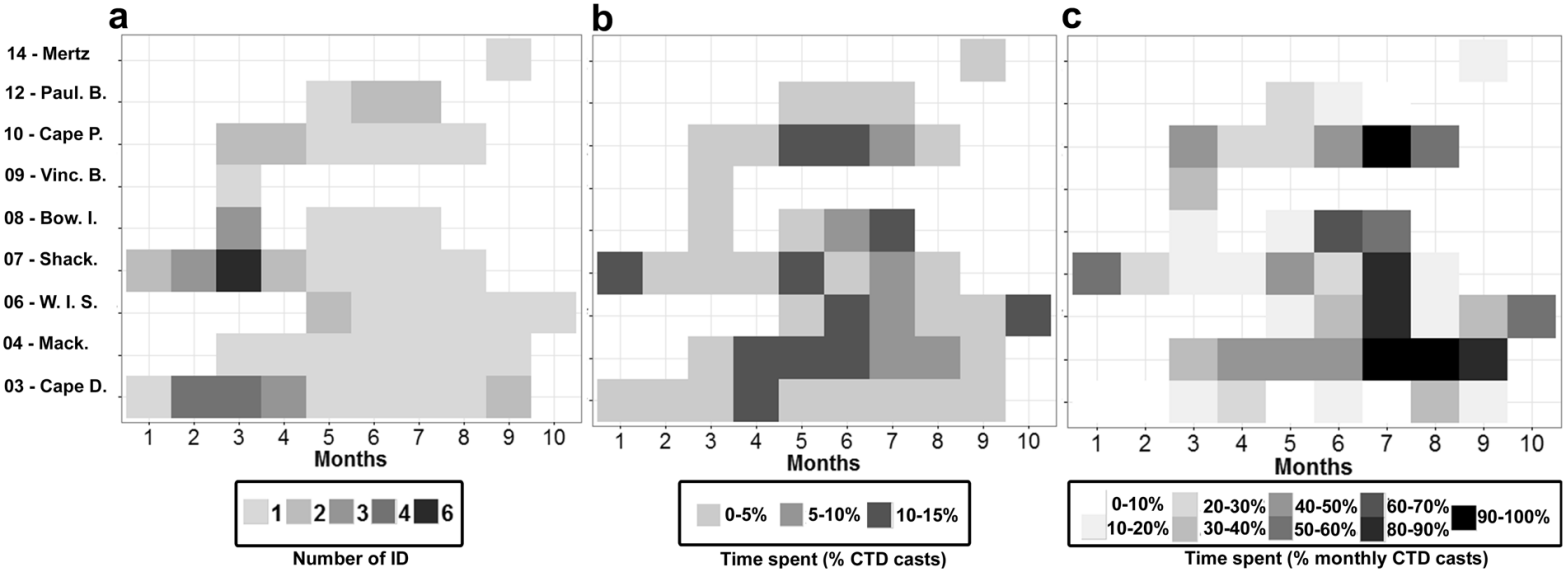
Seasonality of polynya use by the 17 post-moult Kerguelen male SES from 2004 to 2014 based upon the position of CTD casts (n = 1962). Panel (a) represents the number of individuals per month and per polynya, panel (b) the time spent per polynya and per month compared to the whole trip averaged across all seals, and panel (c) the time spent per polynya in a given month averaged across all seals.

Two strategies were observed in how seals used polynyas seasonally (Fig. 1b): half (9 seals) spent short periods (4 to 74 days; with an average across seals of 30 ± 27 days) early in the season (January-March-April) in pre-polynyas, by contrast, the second group (9 seals) remained in polynyas for longer (between 13 days to 190 days and on average 86 ± 57 days) during their post-moult period (February-October). We further examined the seasonality of occupation across the nine polynyas using three metrics: (i) the number of individuals inside polynyas for each month (Fig. 2a); (ii) the percentage of time spent in a given polynya each month compared to the whole trip across all seals (computed per individual and averaged across all seals; Fig. 2b); and (iii) the percentage of time spent in a given polynya each month across all seals (this was also computed per individual and averaged across all seals; Fig. 2c). During the austral autumn-winter (April-September) seals spent inside polynyas; (a) a larger proportion of their trip (Fig. 2c) and (b) a larger proportion of their total time spent in polynyas (Fig. 2b). Five polynyas were used most by seals, i.e. were visited by at least two individuals during the season and where the seals spent 10-15% of their total time, namely: Cape Darley (#3), West Ice Shelf (#6), Shackleton (#7), Bowman Island (#8), and Cape Poinsett (#10).

### Changes in dive and foraging behaviour metrics inside polynyas

**Figure 3:**
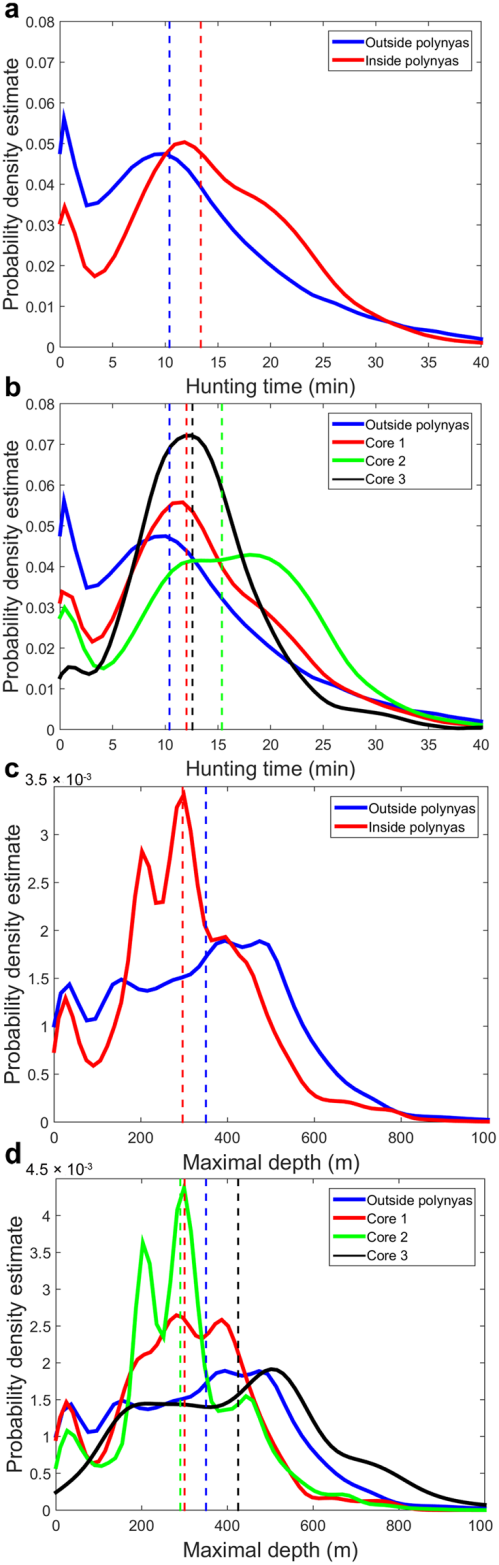
Change in foraging activity (expressed by the hunting time) and diving depth (expressed by maximal depth) inside and outside polynyas and within the different cores of the polynya (defined by SIP_year_ of 2.5, 5 and 10 m.y^−1^, respectively). Panels (a,c) represent the probability density estimate (PDE) inside and outside polynyas respectively and panels (b,d) outside plus inside core areas 1, 2 and 3 respectively. Dashed lines represent the median hunting time for panels (a,b) (expressed in minutes) and the median diving depth for panels (c,d) (expressed in meters). The PDEs were computed based on dive data (n = 18 SES inside polynyas, n = 23 SES outside polynyas). For diving depth, only data on the Antarctic shelf region were used.

Over the seals’ entire trips, hunting time was significantly (i.e. linear mixed effect models) greater inside (13.4 ± 8.6 min per dive; median ± SD) than outside polynyas (10.4 ± 9.7 min; Fig. 3a, Supplementary, Fig. S1a and Table S4). Seals also hunted for significantly longer in polynya cores 2 (15.4 ± 8.9 min (SD)), in comparison to outside polynyas (10.4 ± 9.7 min; Fig. 3b, Supplementary, Fig. S1b, Table S4).

We also examined diving depths inside and outside polynyas, focussing specifically on the Antarctic shelf region since differences in diving depth between oceanic and shelf areas may simply reflect bathymetry variations instead of polynya presence. Over the shelf, seals made significantly shallower dives inside polynyas (296 ± 159 m (SD)) compared to outside (350 ± 201 m; Fig. 3c, Supplementary, Fig. S1c, Table S4). Seals also made significantly shallower dives in polynya cores 1 (300 ± 155 m (SD)) and 2 (290 ± 147 m), compared to outside these cores (Fig. 3d, Supplementary, Fig. S1d, Table S4). Finally, the proportion of benthic dives relative to pelagic dives during the active polynya phase (May-October) was found to increase from 43% to 57% from the core areas 1 to 2. However, benthic and pelagic dives occurred in equal proportions in core areas 3.

Hunting times and diving depths did not differ between core 3 areas (i.e. areas with the greatest sea ice production, SIP_year_ ≥ 10 m.y^−1^) and outside polynyas. However, diving in core areas 3 occurred less frequently, only 6.7% within polynyas. Moreover, only 5 out of the 9 polynyas visited had core 3 areas for at least one year (these are Mackenzie, Cape Darnley, Shackleton, Vincennes Bay and Mertz). Finally, no significant differences were found in dive duration inside and outside polynyas and between the different core areas within the Antarctic shelf region (see Supplementary, Fig. S1e,f, Table S4).

### Oceanographic conditions, seasonal changes and foraging behaviour inside polynyas

Three key water masses layers of the Southern Ocean south of the Polar Front define the principal water masses of the continental slope (see Supplementary, Table S3 for full definitions and acronyms); from the surface to the bottom these are: Antarctic Surface Water (AASW), modified Circumpolar Deep Water (mCDW) and the densest layer Antarctic Bottom Water (AABW, termed modified Shelf Water (mSW) when at depths less than 2500 m). These layers/water mass definitions can be extended onto the shelf region (following refs^34,35^) where mCDW can exist below the AASW if there is a mechanism to bring it onto the shelf.

The AASW layer sits above the pycnocline and is defined by the winter mixed layer (WML, also known as Winter Water) with a seasonal mixed layer at the surface in summer. In coastal polynya regions with sufficient sea ice formation, the WML can reach the ocean bed and so transforms the AASW (and any mCDW) into dense Shelf Water (DSW). DSW is the precursor of the offshore AABW layer. Cold, saline shelf water with insufficient density to form AABW is termed LSSW. LSSW can be simply early season DSW formation that has not had sufficient build-up of salinity from brine-rejection or a mixing between DSW and new intrusions of mCDW in spring/summer after the active convection from sea ice growth has ended. As DSW is exported from the continental shelf and mixes down the slope to form AABW, it is modified, hence the transitional water mass is termed mSW and links DSW on the shelf and AABW below the continental rise. In regions where there is ice shelf interaction, the majority of ISW is produced when the deepest water mass on the shelf (DSW or mCDW) accesses the grounding line and produces glacial meltwater. ISW is the product of positively buoyant glacial meltwater ascending/mixing until it reaches a level of neutral buoyancy, above or within the layer of its source water mass.

**Figure 4:**
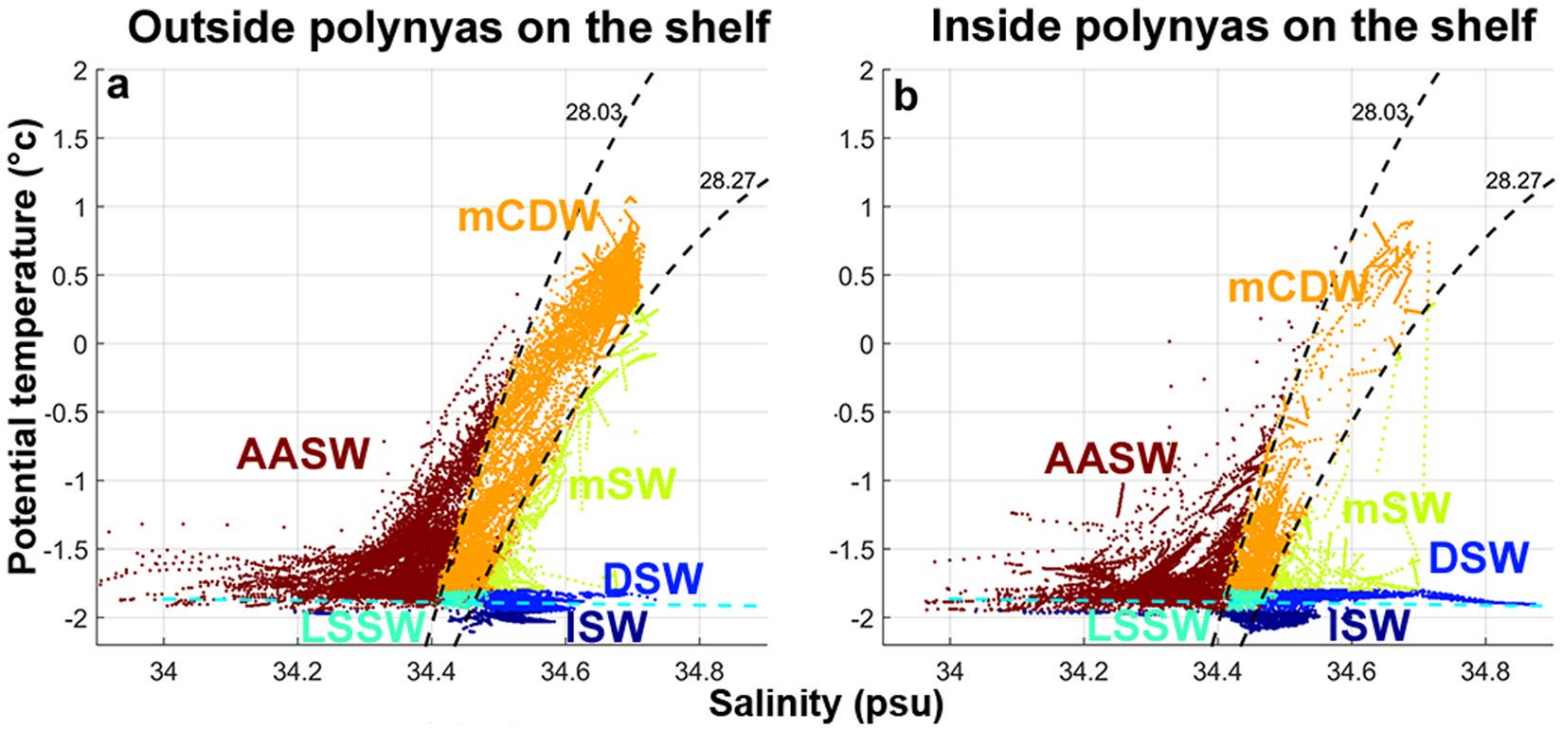
Temperature salinity diagrams of hydrologic properties sampled over the Antarctic shelf from 2004 to 2014 seal CTD casts. Panels (a,b) represent hydrologic properties and water masses outside and inside polynyas sampled at the bottom phase of dives by 19 seals (CTD casts = 1761) and 17 seals (CTD casts = 1844) respectively. Acronyms and definitions of water masses can be found in Supplementary, Table S3.

**Figure 5:**
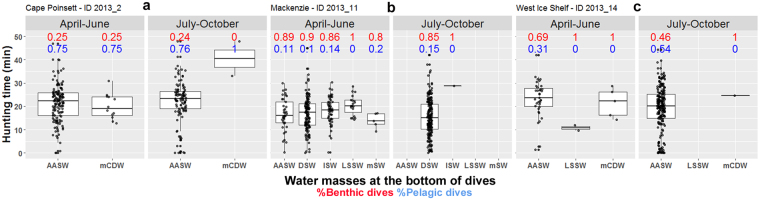
Seal foraging behaviour in relation to water masses for the three the longest time-series available inside polynyas (3 individuals in 2013 visiting the Cape Poinsett (**a**), Mackenzie (**b**) and West Ice Shelf (**c**) polynyas). Water masses sampled at the bottom of dives in relation to the seals’ foraging behavior is represented for the autumn (April-June) and winter season (July-October) in each polynya. A total of 252, 434 and 262 CTD casts were used for Cape Poinsett, Mackenzie and West Ice Shelf polynyas respectively. Black dots represent the observations used to build the boxplots. For each water mass sampled, the proportion of benthic dives is indicated in red while the proportion of pelagic dives in blue.

**Figure 6:**
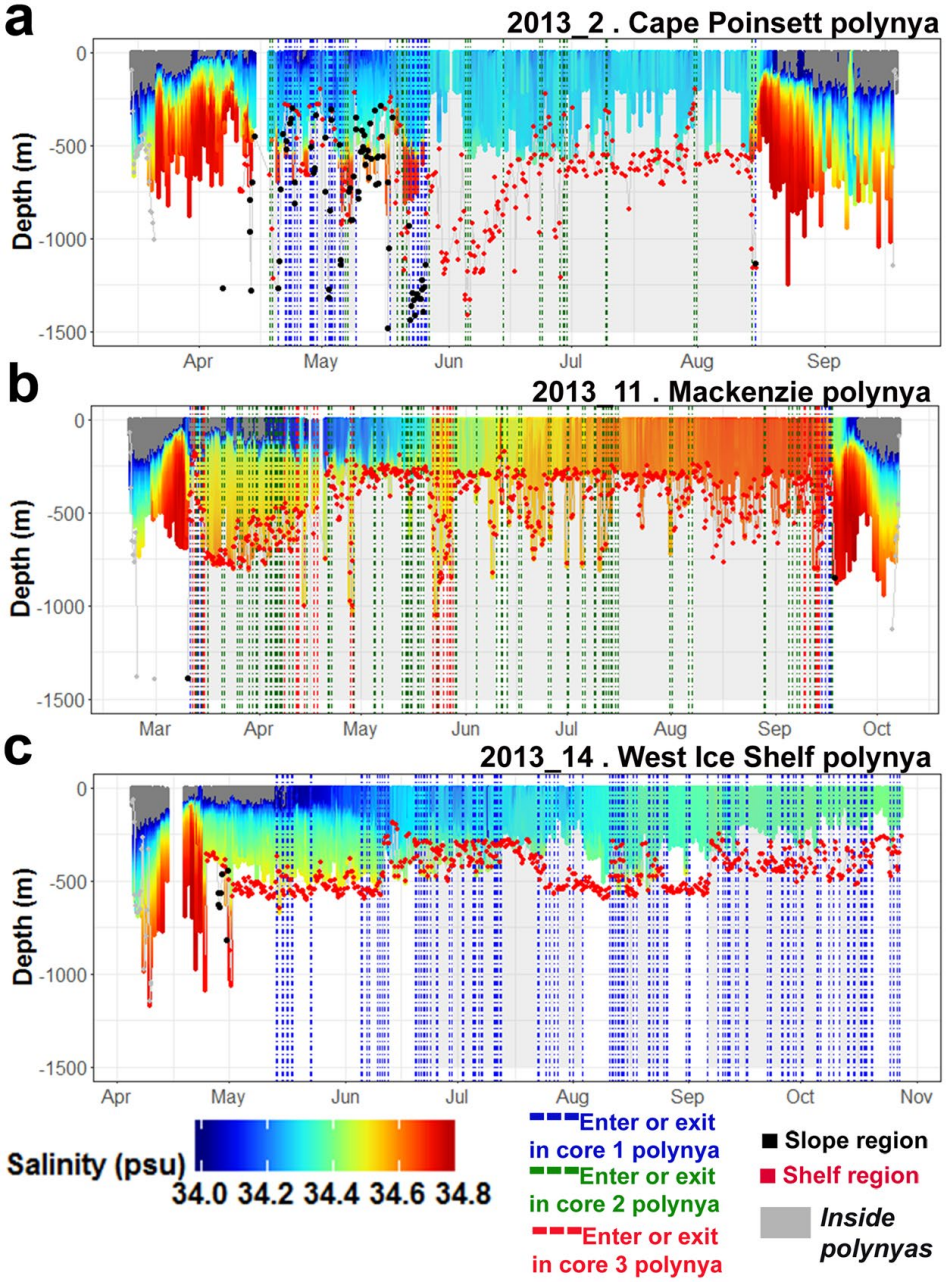
Time-series combining dive information and salinity properties sampled by three individuals in 2013 with the longest time-series available inside polynyas (Cape Poinsett (**a**), Mackenzie (**b**) and West Ice Shelf (**c**) polynyas). The colour displayed on dive profiles corresponds to the salinity (only salinity above 34 is represented, the rest is coloured dark grey). Red and black dots linked by grey lines represent the bathymetry shallower than 1500 m for the Antarctic shelf, slope and outside these regions respectively. Dashed lines correspond to entries and exits of polynyas in the core 1 (2.5 m.y^−1^ of SIP; blue), core 2 (5 m.y^−1^ of SIP; green) and core 3 (10 m.y^−1^ of SIP; red) areas. Seal presence in polynyas is represented by grey shaded on the background.

When on approach to/departure from the shelf region, over the Antarctic slope, the seals encountered AASW, mCDW, and mSW (see Supplementary, Fig. S2). Once on the Antarctic shelf, water masses encountered by the seals are: AASW, mCDW, LSSW, DSW and ISW (Fig. 4a,b). Focusing upon the polynya occupations with the longest time-series available (3 seals visiting each one of the three following polynyas: Cape Poinsett, Mackenzie and West Ice Shelf), we investigated the water masses in relation to the seals’ foraging behaviour (Fig. 5) during both the autumn (April–June) and winter seasons (July–October). We also examined seasonal oceanographic changes inside the polynyas and corresponding seal diving patterns (Fig. 6).

In active coastal polynya regions (e.g. Mackenzie polynya), the autumn period is one of transition, with the remnant summer stratification (summer mixed layer in the AASW layer and mCDW below) being ‘conditioned’ by atmospheric cooling and early sea ice growth. By winter, the entire water column is primed for top-to-bottom convection and increasing salinification of the shelf waters. In contrast, latent heat polynyas near the slope (e.g. Cape Poinsett polynya) have weaker sea ice formation relative to true coastal polynyas and in the absence of true DSW formation, the convection simply deepens the WML within the AASW layer. This deepening AASW layer can reach the bottom in the vicinity of the shelf break.

Seals entered polynyas for the first time between March and May and made successive exits and re-entries (dashed vertical lines, Fig. 6). Among the 3 polynyas, Cape Poinsett polynya is the only one overlapping both the shelf and the slope regions. The seal occupied both regions within this polynya (Fig. 6a; red and black dots) while the two other seals remained exclusively on the shelf (Fig. 6b,c; red dots). During the period from April to May, in all polynyas, the remnant summertime stratification still exists but all seals mainly dived benthically (Fig. 6). At the end of May/beginning of June, a de-stratification in surface waters (~100–200 m) was observed in salinity profiles (Fig. 6). In Cape Poinsett polynya, overlapping the slope region and with weak sea ice production, this period represents the formation of the new WML within the AASW layer. The seal foraged longer in cold, fresh Antarctic Surface Water (Fig. 5a). Within this water mass, the seal mostly dived pelagically, especially during the winter phase (July-October). In contrast, in “true coastal polynyas” with strong sea ice production such as Mackenzie polynya, the period reflects the onset of top-to-bottom convection of the shelf water column. Once Dense Shelf Water formation is occurring, the seal dived all the way to the sea-floor to feed benthically (Fig. 5b). Finally, in West Ice Shelf polynya, a true coastal polynya but with weaker sea ice production compared with Mackenzie polynya, the seal mainly foraged in AASW both pelagically and benthically (Fig. 5c) where the deepening of the AASW layer can reach the bottom.

**Figure 7:**
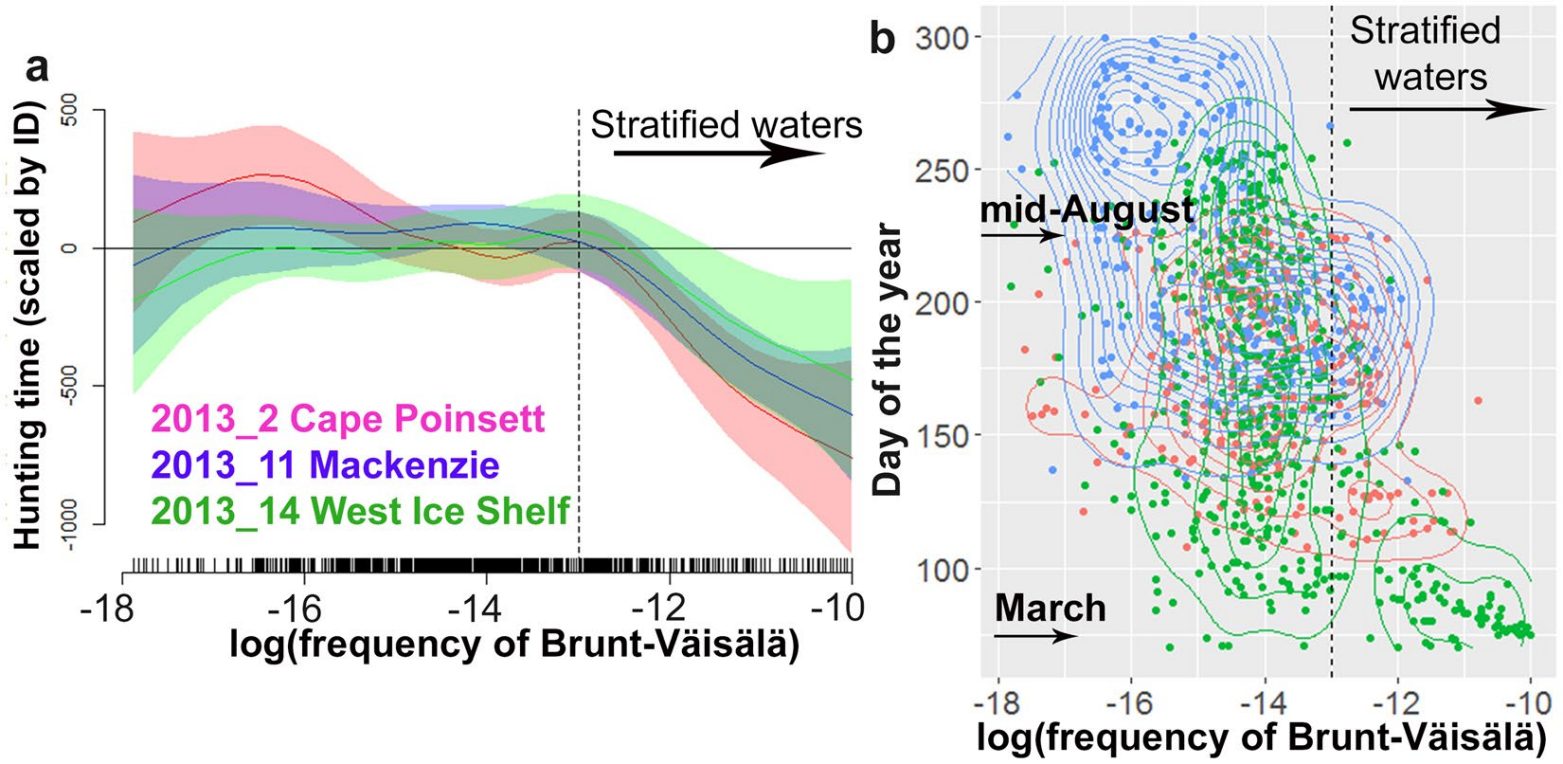
Influence of upper ocean stratification on seal foraging activity for the three individuals in 2013 with the longest time-series available inside polynyas (Cape Poinsett, Mackenzie and West Ice Shelf polynyas). Panel (a) represents fitted relationships from the GAMM relating hunting time to the ocean stratification. Panel (b) represents the density plot distribution of the stratification in function of the season. In both panels, stratification is expressed as the logarithm of the frequency of Brunt-Väisälä. Colour scale represents the three individuals (ID “2013_2” red; “2013_11” blue; and “2013_14” green). For each individual, in panel (a) the thick lines represent the predicted values and the shaded envelopes represent the confidence interval of the predicted values. The vertical dashed lines in panels (a,b) highlight the change point in the fitted relationship and our delineation between less and more stratified waters.

The time-series of salinity were different among the three polynyas in these examples, with respectively, no increase, a slight increase, and a sharp increase in salinity over time in the Cape Poinsett (Fig. 6a), West Ice shelf (Fig. 6c) and Mackenzie (Fig. 6b) polynyas likely reflecting the strength of the polynya in terms of sea ice production. Episodic salinity changes along the winter series may capture brine rejection from sea ice formation, an important determinant of the upper ocean stratification^36,37^. For these seals, a generalized additive model showed that longer dive hunting times occurred in less stratified surface waters (from 10^−18^ to 10^−13^ rad.s^−1^, frequency of Brunt-Väisälä, Fig. 7a). In contrast, a sharp decrease in hunting times was observed for more stratified surface waters (values above 10^−13^ rad.s^−1^, Fig. 7a). Periods of stratification did occur throughout March to mid-August (Fig. 7b).

### No influence of polynya thin ice activity and size on seals’ polynya use and foraging activity

We found no clear link between polynya size (based on polynya sea ice production, i.e. the maximum polynya size defined by 2.5 m.y^−1^ sea ice production) and the daily average of hunting time *per* dive (Supplementary, Fig. S3s). Similarly there was no link between polynya thin ice activity (based on the daily thin ice area scaled by the maximum polynya size) and the daily average of hunting times per dive (Supplementary, Fig. S4).

## Discussion

### General patterns

Our novel observations of seal behaviour coupled with *in situ* oceanographic observations reveals how southern elephant seals use East Antarctic coastal polynyas. This illustrates the seasonal/temporal use and regional occupations of polynyas by high-trophic level predators and thus builds upon and contributes to the body of knowledge of animal behaviour in polynyas. We demonstrated that coastal polynyas are a key winter habitat for subadult males from Iles Kerguelen foraging in East Antarctica, with seals spending up to 75% of their total time at sea occupying polynya region. Among the total SES instrumented in Kerguelen between 2002 and 2017 (males and females combined; n = 354), 56% of the SES foraged in frontal areas of the Antarctic Circumpolar Current (ACC), 44% forage south of 55°S within the seasonal sea ice zone (SSIZ) and 34% inside polynyas (78% of those visiting the SSIZ). This reinforces the importance of both SSIZ and polynyas as SES foraging habitats. Significant changes in the foraging and diving behaviour were evident inside polynyas. Enhanced primary production in coastal polynyas^4,14^, especially diatom blooms, appears to extend feeding and reproduction of secondary producers into late summer and early autumn (reviewed by ref.^9^), resulting in higher resources available inside polynyas during the post-moult period of SES (January – October). This increase in primary and secondary resources may explain the marked increase in seals’ foraging activity in polynyas and the large proportion of time these subadult male seals spent in polynyas during their post-moult foraging trip.

The use of polynyas fell into two strategies over the post-moult period: one group of 9 seals spent relatively short times early in the season (January–April) in pre-polynyas; while the second group of 9 seals remained in polynyas during the pre-polynya phase and the entire winter (February – October). Early in the season, seals of the first group probably took advantage of the enhanced biological activity from spring blooms that took place in post-polynyas^4^, but then left for different foraging areas. Seals from this group may avoid the risk of being trapped in sea ice over winter. Seals from the second group appear to have exploited polynyas throughout the winter period, potentially taking advantage of both the open water access polynyas provide within dense pack/fast ice, and also the prolonged secondary production available here in contrast to surrounding ice covered waters.

### Change in dive patterns and foraging behaviour inside polynyas

The hunting time per dive was used as a proxy for foraging activity. Regarding the validity of this index, findings from the recent work of ref.^38^ on high resolution dive data of SES investigating the link between bottom duration and Prey Encounter Events (PEE) derived from accelerometers were interesting for our study: (i) they showed that for 90% of dives, the bottom time is a good indicator of foraging success (i.e. bottom time increased with the number of PEE at depth greater than 250 m); (ii) they also found that beyond 550 m dive depth, bottom time starts decreasing with increasing dive depth regardless whether or not the dive was successful and unsuccessful (presence or absence of PEE). Hunting time encompasses foraging effort both at the bottom and transit phase and is well correlated with bottom time. The validity of hunting time is thus dependent on diving depth, and below 550 m it may be biased as shorter bottom times (reflecting the physiological dive limits) may be associated with good foraging success. However we found that the average dive depth of male seals foraging within the sea ice region was around 330 m, so this bias may only concern deep dives within canyon along the Antarctic shelf or along the shelf break. We are thus confident that this index is reliable for evaluating foraging activity of SES within the sea ice region.

SES foraging activity was higher and diving depths shallower inside polynyas (core areas 1 & 2) compared with dives performed over the shelf outside polynyas. This pattern presumably reflects the presence of aggregated/abundant prey resources in the upper surface layer in polynyas following extensive primary production in spring. Only a small fraction of dives inside polynyas (6.7%) took place in core 3, and hunting times and diving depths there did not differ markedly from those outside of the polynyas. We suggest that these central zones within the polynyas are likely less available overall (being relatively small areas and not always present in all polynyas).

A bimodal distribution in the diving depths was observed inside polynyas, reflecting the presence in similar proportion of both benthic and pelagic dives (Fig. 3c and Supplementary, Table S1). The proportion of benthic dives relative to pelagic dives during the active polynya phase (May–October) was found to increase from 43% to 57% from the core areas 1 to 2. We suggest that SES may switch from dominant pelagic to benthic feeding when increased sea ice production inside polynyas leads to mixing of shelf waters. Prey distribution within the water column may change from stratified to well-mixed shelf waters. For example, within the massive Ross Sea polynya, the inverted marginal ice zone ringing the polynya (i.e. associated with lower sea ice production and lower mixing of the shelf waters) may harbour higher quantities of fish and krill within the water column^39^ than the polynyas’ central waters as it often harbours more diatoms compared to central mixed waters^12,40–42^. In contrast, well mixed shelf waters may harbour rich benthic communities benefiting from enhanced vertical carbon flux^10^. Other parameters varying from one polynya to another such as intrusion of nutrient rich mCDW and bottom depth may also influence the switch in seal diving behaviour. Below we develop concepts on the underlying oceanographic mechanisms coupling primary production, secondary producers and predators.

### Oceanographic conditions, seasonal changes and foraging behaviour in polynyas

**Figure 8:**
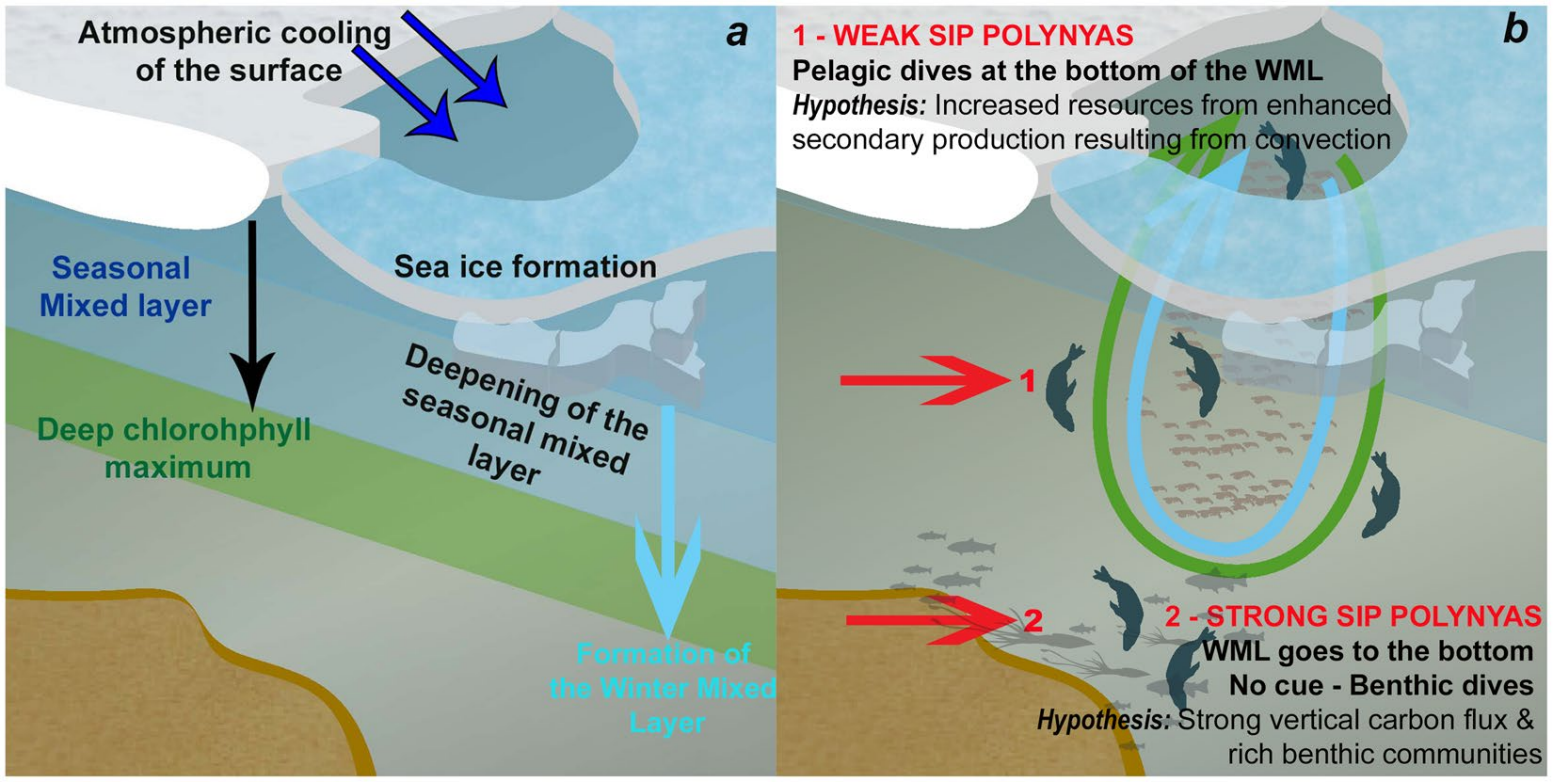
Schematic summarising how seasonal changes in autumn/winter in the structure of the water column may change resource distribution and influence seal foraging activity in different ways within each polynya (expanded from ref.^43^). The illustration was made by S. Labrousse using Adobe Illustrator.

Water column de-stratification occurs during autumn-winter as brine rejection associated with sea-ice formation increases salinity (Fig. 8a). In terms of seal behaviour, de-stratified waters were associated with the longest hunting times inside polynyas. Ref.^43^ reported similar behaviour in post-moulting female SES from Macquarie Island and foraging in the Commonwealth Bay polynya (66.5–67°S, 142–143.5°E) during the summer-autumn transition (i.e. Feb–April). The authors hypothesized that the continuous foraging activity they observed was likely due to favourable feeding conditions resulting from ongoing secondary production interpreting their observations as follows. In summer, the shallower seasonal mixed layer forms above the winter mixed layer, in some cases in conjunction with a deep chlorophyll maximum. After summer, atmospheric cooling initiates re-freezing, sea ice growth and the development of the new winter mixed layer (Fig. 8a). The deepening of the winter mixed layer, via wind and sea ice formation, can entrain nutrients from below, and remix the subsurface bloom. Such a mechanism could enhance secondary production through trophic cascades during autumn^44,45^. The longer hunting times we reported were also associated with de-stratified waters inside the three polynyas and similarly likely represent SES taking advantage of enhanced secondary production resulting from these mixing processes, especially during pelagic dives (Fig. 8b).

The three polynyas studied have different oceanographic structure and properties and in accordance seal foraging behaviour varied among the three polynyas. Mackenzie polynya is the polynya among the three, where active sea ice formation allows the formation of DSW. Here, the WML convects all the way to the sea-floor. Within this polynya, seals mostly visited shelf waters in autumn and then dived only benthically within dense shelf waters in winter. Mooring data in that location suggests convection to 1000 m in winter^46,47^. Overlying a deep depression (700 m to 1200 m deep with a diameter of ~10 km), the Mackenzie polynya is isolated from the rest of Prydz Bay below depths of about 700 m and may harbor deep prey for SES such as cephalopods. Indeed, the abundant Antarctic squids *Galiteuthis glacialis* and *Psychroteuthis glacialis* are restricted to cold Antarctic waters^48^. We hypothesized that these species may inhabit the Mackenzie depression as adults of *G*. *glacialis* were found to be concentrated in the 800–1000 m layer (mainly beyond the shelf break) and adults of *P*. *glacialis* were found near the bottom close to the shelf break within the Prydz Bay region^49^. The West Ice Shelf polynya has only minor sea ice production and it appears that the seals dived both pelagically and benthically in AASW, presumably chasing the prey aggregated at the boundary of the Winter Mixed layer that can sometimes reach the bottom. Finally, Cape Poinsett is mostly formed due to divergence in the westward flow of ice with the coastal current. There is an important presence of mCDW at depth in that region; any deep convection may cause heat to come to the surface, stopping sea ice production, but possibly assisting in keeping the polynya open. Our results show that in autumn and winter, the seals mainly hunted pelagically in the AASW, most likely chasing the prey aggregating near the boundary of the WML as seen in the West Ice Shelf polynya.

To conclude, we suggest that during the autumn-winter (April to October), in low sea ice production polynyas, seals dived pelagically to reach the bottom of the WML, remaining in the AASW, where they may benefit from increased resources from convection (Fig. 8b). In contrast, in strong sea ice production polynyas, the WML extends to the sea-floor, resulting in an homogeneous water column with no pycnocline concentrating prey in its vicinity, and which may drive the seals to forage on the benthos (Fig. 8b). Polynyas with strong sea ice production may be associated with strong vertical carbon flux to the sea-floor^10^ and specific bathymetric features which then lead to rich benthic communities and prey.

### No influence of polynya size and variability on seals’ polynya use and foraging activity

Polynya size, nor its variability in terms of thin ice area influenced seals’ usage of polynyas or their foraging activity. While marine mammals and birds are reportedly more abundant in large and productive polynyas^4,12,20^, the mechanism by which polynya size enhances productivity remains unclear. As detailed by ref.^14^, polynya size does not directly influence primary production via (i) mixed layer depth (as mixed layer depths in polynyas of different sizes are similar), (ii) nutrient availability, (iii) sea ice melt releasing iron (as sea ice can also be advected away by winds and currents), or (iv) light availability (as there is only a weak relation between open water area and photosynthetically usable radiation). Further studies are needed to establish linkages between polynya size, productivity and implications across trophic levels within polynya ecosystems. Moreover, all polynyas are different in terms of their physical and biological capabilities.

### Conclusion and perspectives

Using seven years of tracking, we provide clear evidence that subadult male SES from Iles Kerguelen that forage in East Antarctica during their post-moult trip use coastal polynyas to feed. Both pre-polynyas during late summer and active polynyas from autumn through the end of the winter season were used by seals, and foraging activity increased significantly inside polynyas. Unique biological and physical features characterize polynyas as winter oases for these marine predators: (i) open water access to breathe at the surface throughout the winter; (ii) secondary production in autumn from mixing associated with the formation of the WML and water column de-stratification; and (iii) rich benthic communities from likely enhanced vertical water column mixing and carbon flux in strong sea ice production polynyas.

It remains unclear why some polynyas were used more than others. Inter-polynya differences in oceanographic conditions, topography, proximity to the Antarctic Slope Front, and sea ice conditions may account for some of the differences in seal behaviour we observed. However, seals may also choose polynyas through innate behaviours, *e*.*g*. opportunistic feeding when high prey patches are present, or predator avoidance and thus may remain in the polynyas as sea ice extends for the whole winter. The role of innate behaviours such as opportunism, predator avoidance or the role of memory is difficult to unravel when analyzing the foraging ecology of predators. Although this study was based on a unique bio-physical dataset recorded by southern elephant seals across 11 years representing an important research effort, our conclusions are still limited by a relative small sample size (n = 23) leading to potential high degree of individual variability in foraging behavior.

Finally, given the spatio-temporal complexity of polynya processes, it is worth considering how these key habitats might respond to climatic changes. Phytoplankton biomass and primary production in coastal polynyas may increase in the future as stirring of sediments, sea ice and Antarctic ice shelf melt release iron, which may explain some of the variance in primary production in polynyas^50^. Conversely, physical drivers of vertical mixing important for ecological processes may be slowed or shut down in weaker polynyas. Thus, the ecological importance of key coastal polynyas may change for meso-predators that rely on them as a foraging ground. Understanding how meso-predators rely on particular sea-ice features deepens our much-needed knowledge on under-ice biological habitats. Indeed, knowing how environmental structure and dynamics affect productivity is central to understand the ecology of the Southern Ocean and provides the basis for predicting consequences of future changes on this unique ecosystem.

## Methods

### Tagging and behavioural data

We used satellite location and dive depth data from 23 post-moulting Kerguelen subadult male SES (weight: 559 ± 244 kg (SD); Supplementary, Table S2) that were instrumented with Conductivity-Temperature-Depth Satellite Relay Data Loggers (CTD-SRDLs, Sea Mammal Research Unit, University of St Andrews) between December and February in 2004, 2008–2009 and 2011–2014 at Kerguelen Islands (49°20’S, 70°20’E). These animals were chosen from a larger dataset because they visited the Southern Ocean region south of 55°S (the spatial domain for the study), which corresponds to the maximum latitude of annual sea ice extent. This dataset comprises a total of 136,599 transmitted dives, 8568 CTD casts and 69,479 ARGOS satellite locations across all animals (Supplementary, Table S2). Full details on instrumentation, seal handling and data processing for dives, ARGOS position filtering and CTD data treatment are provided in ref.^26^. All animals in this study were handled in accordance with the French Polar Institute (Institut Paul Emile Victor, IPEV) ethical and Polar Environment Committees guidelines. The experimental protocols were approved by The Ethics Committee of IPEV and Polar Environment Committees.

The proxy for foraging activity for each seal was developed at the dive scale using the methodology developed by ref.^51^, which estimates the time spent hunting during a dive. For each dive, the time spent in segments with a vertical velocity lower or equal to 0.4 m.s^−1^ was calculated. This time was the estimated hunting time per dive and was used as a proxy for foraging activity.

For each individual, the time spent within or outside polynyas, in the different polynya core areas (defined below), spatial zones (shelf, slope and open ocean) and using benthic or pelagic diving strategies were calculated from the dive duration of the dive data (Supplementary, Table S1). The exceptions were individuals 2011_4 and 2011_9 (marked with a star in Table S1), for which the dive data stopped recording in May and the CTD data (which continued until September) were used instead. For these individuals, the calculations were computed using the number of CTD observations in each case divided by the total number of CTD observations. For consistency between individuals, Figs 1 and 2 were based on time spent computed from CTD casts for all individuals. CTD casts represent the two deepest dives an animal has undertaken in each 6 h period (since the tag CTD protocol is designed to maximise observational coverage of the water column).

### Polynya identification

Polynya location and extent was based on estimated sea ice production (expressed in m.y^−1^) following refs^52–54^ using updated data from ref.^3^. First, the thin ice thickness algorithm was applied using 85 and 37 GHz brightness temperature retrieved from SSM/I. Next, sea ice production was estimated by heat flux calculation during the freezing period (from March to October) using thin ice thickness and surface atmospheric data. The air-sea-ice surface heat flux is obtained by assuming that the sum of radiative and turbulent fluxes at the ice surface is balanced by the conductive heat flux in the ice. The European Centre for Medium-Range Weather Forecasts Re-Analysis data (ERA-40: 1992–2001, ERA-interim: 1992–2014) and the National Centres for Environmental Prediction/Department of Energy Re-Analysis data (NCEP2: 1992–2014) were used for this calculation. The calculation was performed twice a day over the entire Southern Ocean on the SSM/I Equal Area Scalable Earth-Grid (12.5 km X 12.5 km) from 1992 to 2014.

**Figure 9:**
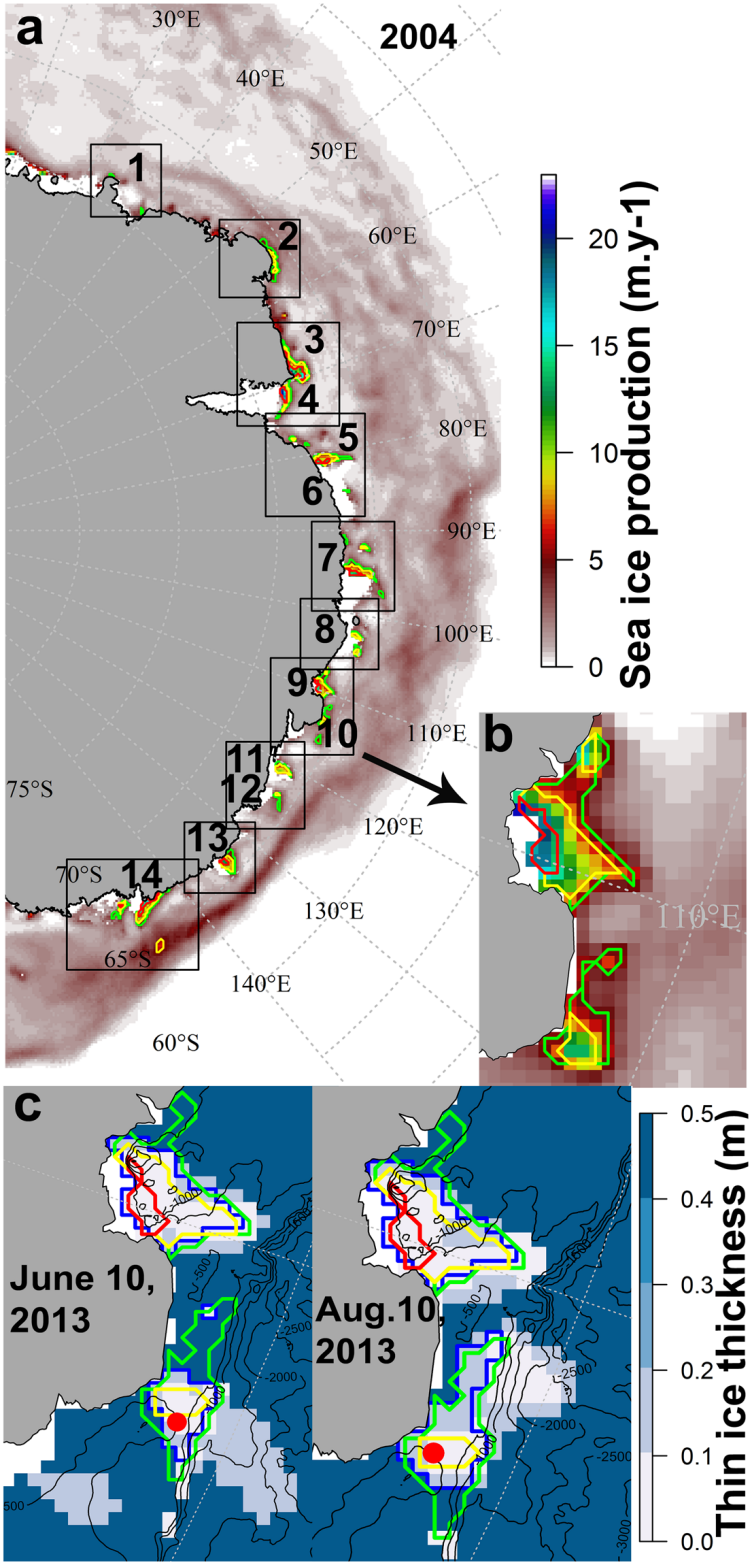
Illustration of the polynya definition based on sea ice production and thin ice thickness. Panel (a) represents an example of annual SIP (m.y^−1^) for 2004 obtained from the sum of monthly SIP during the freezing period from March to October. Boxes pre-defining polynya areas were based on literature, within which polynya polygons were computed based on threshold of SIP_year_ of 2.5 m.y^−1^ (green contour), 5 m.y^−1^ (yellow contour) and 10 m.y^−1^ (red contour). A total of 14 polynyas were identified: 1. Lützoh-Holm Bay, 2. Cape Borle, 3. Cape Darnely, 4. Mackenzie, 5. Barrier, 6. West Ice Shelf, 7. Shackleton, 8. Bowman Island, 9. Vincennes Bay, 10. Cape Poinsett, 11. Dalton, 12. Paulding Bay, 13. Dibble, 14. Mertz. The inset (b) is a zoom of polynyas 9 and 10 to highlight the different SIP contours. Panel (c) represents example maps showing daily thin ice thickness data for two dates during 2013. SIP contours are coloured as in panel (b). Black contours represent bottom topography. Within the largest polynya area based on 2.5 m.y^−1^ SIP (green polygon), daily polygons were then computed for ice thickness between 0 and 0.2 m (blue contour). The red dot corresponds to one seal position for each given date. The maps were made by S. Labrousse using R software, version 3.2.4 revised (R Core Team (2016). R: A language and environment for statistical computing. R Foundation for Statistical Computing, Vienna, Austria. https://www.R-project.org/). The bathymetry contours represented in panel (c) are from The GEBCO_08 Grid, a global 30 arc-second grid largely generated by combining quality-controlled ship depth soundings with interpolation between sounding points guided by satellite-derived gravity data. http://www.gebco.net/data_and_products/gridded_bathymetry_data/. The legal copyright of the main components of the GEBCO Digital Atlas is held by the UK Natural Environment Research Council (NERC) on behalf of the Intergovernmental Oceanographic Commission (IOC) of UNESCO and the International Hydrographic Organization (IHO), through the Joint IOC/IHO Guiding Committee for GEBCO.

The determination of polynyas was then based on sea ice production thresholds following the steps presented in Supplementary, Fig. S5. We first determined the annual sea ice production for each polynya, SIP_year_, by summing monthly sea ice production during the freezing period (step 1, Supplementary, Fig. S5). Then, for efficiency we defined spatial boxes around known polynya regions based on the published literature^4,14,17,55^. Within these boxes, we drew three polygons to define polynya regions based on SIP_year_ corresponding to 2.5 m.y^−1^ ≤ SIP_year_ < 5 m.y^−1^ (“core 1 or outer core”, green contour), 5 m.y^−1^ ≤ SIP_year_ < 10 m.y^−1^ (“core 2 or middle core”, yellow contour) and SIP_year_ ≥ 10 m.y^−1^ (“core 3 or inner core”, red contour) (Fig. 9a,b; step 2a and 2b, Supplementary, Fig. S5). For each polynya, based on daily estimates of thin ice thickness, within the largest polygon (2.5 m.y^−1^ of SIP_year_) we contoured a polygon of thin ice (“daily cores”, characterized by a thickness from 0 to 0.2 m, blue contour; Fig. 9c, step 3, Supplementary, Fig. S5). This characterized the variability of the distribution of thin ice from one day to another inside the area defined by SIP_year_. The three step procedure for defining polynyas is illustrated by an example for 2004 (Fig. 9). A total of 14 polynyas were identified and named following refs^4^ and^17^: 1. Lützoh-Holm Bay, 2. Cape Borle, 3. Cape Darnley, 4. Mackenzie, 5. Barrier, 6. West Ice Shelf, 7. Shackleton, 8. Bowman Island, 9. Vincennes Bay, 10. Cape Poinsett, 11. Dalton, 12. Paulding Bay, 13. Dibble, 14. Mertz. For each of the 14 polynyas, we defined a mean annual position of the polynya, based on the three thresholds corresponding to the three “cores” of the polynya and the “daily core” region as detailed above. The area of each polygon (expressed in squared metres) was computed (step 4, Supplementary, Fig. S5). The shortest distance between each seal position and each polygon contour calculated (step 5, Supplementary, Fig. S5) and whether each seal position was inside/outside of each polygon determined (step 6, Supplementary, Fig. S5).

### *In situ* salinity/temperature profiles and water mass definition

Among the 23 male SES, 21 had usable CTD (Conductivity–Temperature–Depth) profiles for a total of 8568 profiles (Supplementary, Table S2). All tags were initially calibrated at the laboratory and a proportion of them (35%) were also tested at sea against a ship based CTD before deployment. All tags were post-calibrated^56,57^. The minimum accuracies of post processed data were estimated to be ±0.03 °C in temperature and ±0.05 psu, increasing to ±0.01 °C and ±0.02 psu in the best cases^57^. Data are made freely available by the International MEOP consortium (Marine Mammals Exploring the Oceans Pole to pole, http://www.meop.net) and the national programs that contribute to it.

To obtain temperature and salinity vertical profiles at regularised depths, a linear interpolation every 5 m was applied while the average depth difference between points of each profile among all individuals were 35 ± 45 m (mean ± SD). CTD positions were corrected by interpolating between filtered locations at the CTD timestamp. For each CTD profile, we identified the water masses used when the seals were at the dive bottom, as this is where most of the foraging activity is expected to occur^58^. The start of the bottom phase was defined as 80% of the maximal depth of the CTD profile^59^. To associate temperature and salinity profiles (average of 2.8 ± 1 (SD) profiles per day, n = 21) with foraging activity and dive parameters (*e*.*g*. dive duration) each CTD profile was compared with the closest dive in time and depth collected by the same individual as follows: for each CTD profile only (i) dives with a maximum depth shallower or equal to the CTD depth, and (ii) within ±6 hours of the CTD timestamp were retained; (iii) from this selection, the dive with the closest depth was retained; (iv) if more than one dive was retained from the previous selection, the closest in time was selected. The stratification within the upper 100 meters of the water column has been computed as the frequency of Brunt-Väisälä, ${N}^{2}=g/{\rho }_{0}\cdot \partial {\sigma }_{0}/\partial z$, where ${\rho }_{0}$ = 1000 kg.m^−3^, *g* is the gravitational acceleration of the Earth ($g$ ~ 9.81 m.s^−2^), and ${\sigma }_{0}$ is the surface-referenced potential density. Namely, for each profile, we computed ${N}_{app}^{2}=g/{\rho }_{0}\cdot ({\sigma }_{0}(100\,m)-\,{\sigma }_{0}(0m))/100\,m$ which is an approximation of the frequency of Brunt-Väisälä, representing the depth-average of ${N}^{2}$ over the upper 100 m of the water column.

Water masses sampled during the transit of seals from 55°S to the Antarctic continent were determined from their temperature, salinity and neutral density *y*_*n*_ characteristics^60^, in conjunction with some specific geospatial conditions. Criteria to define these water masses (Supplementary, Table S3) were adapted from refs^26,28^, following the literature^34,35,61–65^. We distinguished eight major water masses: (1) Antarctic Surface Water (AASW); (2 & 3) Circumpolar Deep Water (CDW) and modified CDW (mCDW); (4) modified Shelf Water (mSW), north and south of the shelf break; (5) Antarctic Bottom Water (AABW); (6) Ice Shelf Water (ISW); (7) Dense shelf water (DSW); (8) Low Salinity Shelf Water (LSSW).

### Statistical analysis

To quantify the influence of (i) polynyas on seal foraging activity, a total of 6 linear mixed effects models (LMMs) were fitted with the R package *nlme*^66^ using restricted maximum likelihood (REML). The 6 different models tested the difference in hunting times, dive duration and maximum depths inside and outside polynyas (models 1, 2, 3); in the 3 different yearly cores and outside polynyas (models 4, 5, 6). Models were based on dive data on a total of 23 males (taking one dive every three dive to allow model computation).

For each model, the response variable was centred and scaled for each seal prior to analysis to correct for non-Gaussian distribution. We first determined the optimal model structure by assessing if individual seals as a random intercept term contributed to the model fit. The final model was then fitted using restricted maximum likelihood (REML). Model validation was checked by plotting Pearson residuals against fitted values, and against the explanatory variables, to verify homogeneity and normality of residuals^67^. The significance of each factor relative to the first level of factor was assessed using p-values with a threshold of 0.01. Summary of the regression coefficients for the 6 LMMS are presented in Supplementary, Table S4 and Fig. S1.

Finally, a Gaussian additive mixed effects model (GAMM) was fitted to examine the statistical relationships between seal hunting time per dive and the ocean stratification inside polynyas. These focussed upon the three longest time-series available inside polynyas (3 seals visiting each one of the three following polynyas: Cape Poinsett, Mackenzie and West Ice Shelf). The model was computed with the R packages *itsadug* (ref.^68^; function *gam*). Outliers in the explanatory variables were checked. Individual seals were included as a random intercept and slope terms. Model validation and term significance were evaluated as described above. Summary of the regression coefficients for the GAMM are presented in Supplementary, Table S5.

### Data availability

The datasets analysed and generated during the current study are available from the corresponding author on reasonable request.

## Electronic supplementary material

Supplementary Materials
