# Supplementary material for: Coastal polynyas: Winter oases for subadult southern elephant seals in East Antarctica
Source: Sci Rep. 2018 Feb 16;8:3183. doi: 10.1038/s41598-018-21388-9 (PMC5816617; doi:10.1038/s41598-018-21388-9)
Supplement: Supplementary file 1 — Supplementary Materials [file 41598_2018_21388_MOESM1_ESM.pdf]

Coastal polynyas: Winter oases for subadult southern elephant seals in East Antarctica

Labrousse, Sara[1,2]; Williams, Guy[2,3]; Tamura, Takeshi[3,4,5] ; Bestley, Sophie[2,3,6] ; Sallée, Jean-Baptiste[1,7]; Fraser, Alexander D.[3,8]; Sumner, Michael[3,6]; Roquet, Fabien[9]; Heerah, Karine[1,2]; Picard, Baptiste[10]; Guinet, Christophe[10]; Harcourt, Robert[11] ; McMahon, Clive[2,11,12]; Hindell, Mark A.[2, 4]; Charrassin, Jean-Benoit[1].

(1) Sorbonne Universités, UPMC Univ., Paris 06, UMR 7159 CNRS-IRD-MNHN, LOCEAN-IPSL, 75005 Paris, France

(2) Institute for Marine and Antarctic Studies, University of Tasmania, Private Bag 129, Hobart, Tasmania 7001, Australia

(3) Antarctic Climate & Ecosystems Cooperative Research Centre, University of Tasmania, Private Bag 80, Hobart, Tasmania 7001, Australia

(4) National Institute of Polar Research, 1-9-10, Kaga Itabashi-ku, Tokyo 173-8515, Japan

(5) SOKENDAI (The Graduate University for Advanced Studies), Tachikawa, Tokyo 190-8518, Japan

(6) Australian Antarctic Division, Channel Highway, Kingston, Tasmania 7050, Australia

(7) British Antarctic Survey, High Cross, Cambridge, CB3 0ET, UK

(8) Institute of Low Temperature Science, Hokkaido University, N19 W8, Kita-ku, Sapporo 060-0819, Japan

(9) Department of Meteorology, Stockholm University, S-106 91 Stockholm, Sweden

(10) Centre d'Etudes Biologiques de Chizé (CEBC), UMR 7372 Université de la Rochelle-CNRS, 79360 Villiers en Bois, France

(11) Department of Biological Sciences, Macquarie University, Sydney, New South Wales 2109, Australia

(12) Sydney Institute of Marine Science, 19 Chowder Bay Road, Mosman, New South Wales 2088, Australia

Supplementary Information

Table S1. General information on the 18 post-moulting Kerguelen male SESs that visited polynyas from January to November 2004 to 2014 including year, weight, time spent in polynyas based on dive durations, the number of entries and exits in/out of the polynyas, time spent in polynyas when the seal was on shelf/slope region, and information on the behaviour inside the polynyas: time spent on the shelf, time spent on the slope, time spent in thin ice, time spent in core 1 (2.5 $m.y^{-1}$ SIP), time spent in core 2 (5 $m.y^{-1}$ SIP), time spent in core 3 (10 $m.y^{-1}$ SIP), benthic dives, pelagic dives, pelagic dives on slope, pelagic dives on shelf, benthic dives on slope, benthic dives on shelf. Averages are expressed ± Standard Error of the Mean (SEM). Statistics are computed from the dive data except for individuals 2011_4 and 2011_9 (marked with a star) where CTD data were used instead because the dive data stopped recording at-sea in May while the CTD continued until September. Individuals underlined in bold italics are individuals for which the tag did not stopped before they returned to the colony.

Seal ID	Year	Weight (kg)	Time spent in polynya over the whole trip (%)	Number of enter/exits	Time spent in polynyas when on the shelf region (%)	Time spent in polynyas when on the slope region (%)	**Time spent on the shelf** when the seal is inside polynyas (%)	**Time spent on the slope** when the seal is inside polynyas (%)	**Time spent in thin ice (0-0.2 m)** when the seal is inside polynyas (%)	**Time spent in core 1 (2.5 m/y of SIP)** when the seal is inside polynyas (%)	**Time spent in core 2 (5 m/y of SIP)** when the seal is inside polynyas (%)	**Time spent in core 3 (10 m/y of SIP)** when the seal is inside polynyas (%)	**Benthic dives** when the seal is inside the polynyas (%)	**Pelagic dives** when the seal is inside the polynyas (%)	**Pelagic dives** when the seal is inside the polynyas **on the slope** (%)	**Pelagic dives** when the seal is inside the polynyas **on the shelf** (%)	**Benthic dives** when the seal is inside the polynyas **on the slope** (%)	**Benthic dives** when the seal is inside the polynyas **on the shelf** (%)
2004_2	2004	385,5	21	125	67	17	45	55	23	94	6	0	29	71	75	66	25	34
2004_5	2004	469,5	27	62	56	**0**	100	0	21	50	50	0	100	0	0	0	100	100
2004_8	2004	274	6	69	15	**0**	100	0	13	100	0	0	24	76	_	76	_	24
2008_1	2008	266	22	89	56	1	99	1	94	93	7	0	82	18	100	18	0	82
2011_4*****	2011	800	45	40	60	**0**	100	0	47	35	21	44	68	32	_	32	_	68
2011_7	2011	452,5	15	18	21	9	93	7	63	33	47	20	83	18	47	15	53	85
2011_9*****	2011	628,5	21	88	59	15	58	42	45	56	22	22	74	26	26	26	74	74
2012_1	2012	523	9	36	32	3	78	22	50	54	44	2	65	35	46	32	54	68
2012_2	2012	303	52	22	77	**26**	93	7	46	67	27	7	94	6	41	4	59	96
2013_2	2013	1100	55	52	**94**	13	96	4	67	25	75	0	16	84	70	84	30	16
2013_3	2013	468	2	**4**	**9**	**0**	100	0	9	79	21	0	72	28	_	28	_	72
2013_9	2013	470	12	10	35	**0**	100	0	48	77	23	0	75	25	_	25	_	75
2013_11	2013	556	**75**	114	88	**0**	100	0	67	9	85	5	79	21	_	21	_	79
2013_13	2013	600	**4**	8	12	**0**	100	0	78	91	9	0	66	34	_	34	_	66
2013_14	2013	300	36	**214**	43	**0**	100	0	14	100	0	0	53	47	_	47	_	53
2014_7	2014	405	**4**	**4**	12	**0**	100	0	78	14	46	40	0	100	_	100	_	0
2014_9	2014	700	22	26	37	**0**	100	0	12	64	31	5	0	100	_	100	_	0
2014_10	2014	700	26	54	63	**0**	100	0	62	47	54	0	0	100	_	100	_	0
Average (SEM)		522 (212)	25 (20)		46 (27)	5 (8)	92 (16)	8 (16)	47 (26)	60 (29)	32 (25)	8 (14)	54 (34)	46 (34)	51 (31)	45 (34)	49 (31)	55 (34)

Table S2. General information of the 23 post-moulting Kerguelen male SESs. It includes dive start and end date, date of return when the tag did not stopped, animal weight and snout-to-tail length upon deployment, if the CTD (Conductivity-Temperature-Depth) data were usable for analysis, number of Argos position transmitted daily, total number of dives, mean number of dives per day and mean distance travelled per day between the first and last locations of each day, if the animal visited polynyas during its trip, total number of CTD profiles, and number of CTD profile per day. Mean are expressed ± Standard Deviation.

ID	Dive start date	Dive end date	Date of return to the colony	Weight (kg)	Length (cm)	Functional CTD	Number of positions transmitted daily	Total dives	Daily number of dives	Distance travelled per day (km)	Inside polynyas	Number of CTD profiles per day	Total CTD
2004_1	04/03/2004	29/03/2004		368	250	No	8 ± 4	553	25 ± 12	75 ± 49	No		
2004_2	27/02/2004	09/07/2004		385,5	267	x	18 ± 7	6133	46 ± 20	34 ± 33	x	2,9 ± 1	336
2004_5	25/02/2004	06/08/2004		469,5	282	x	17 ± 6	7209	46 ± 18	22 ± 31	x	2,7 ± 1	371
2004_8	27/02/2004	08/08/2004	6 then South	274	235	x	17 ± 9	7530	50 ± 25	40 ± 34	x	2,5 ± 1	343
2008_1	01/01/2008	08/09/2008		266	230	No	9 ± 5	8815	39 ± 30	33 ± 26	x		
2009_16	01/01/2009	03/06/2009	6	258	249	x	17 ± 7	5887	40 ± 18	34 ± 28	No	1,9 ± 0,3	266
2011_1	27/01/2011	20/02/2011		680	316	x	21 ± 7	1002	40 ± 13	98 ± 30	No	1,8 ± 0,4	42
2011_4	31/01/2011	16/05/2011		800	330	x	26 ± 7	4438	42 ± 11	33 ± 39	x	1,9 ± 0,4	411
2011_7	26/01/2011	15/04/2011		452,5	280	x	34 ± 10	4749	60 ± 19	36 ± 39	x	1,7 ± 0,5	104
2011_9	27/01/2011	16/05/2011		628,5	326	x	18 ± 6	3487	32 ± 12	29 ± 37	x	1,8 ± 0,4	407
2012_1	23/01/2012	14/09/2012		523	291	x	18 ± 6	9799	43 ± 18	31 ± 28	x	3,2 ± 0,8	737
2012_2	23/01/2012	26/04/2012		454	277	x	24 ± 6	4297	45 ± 11	36 ± 38	x	3,4 ± 0,8	321
2013_2	08/03/2013	02/11/2013	11	1100	370	x	17 ± 10	8321	39 ± 17	33 ± 41	x	3,3 ± 0,8	627
2013_3	10/02/2013	17/03/2013		468	280	x	22 ± 9	1513	46 ± 9	67 ± 41	x	3,2 ± 0,8	105
2013_4	03/03/2013	09/09/2013	9	850	333	x	18 ± 7	6064	35 ± 12	36 ± 36	No	3 ± 1	560
2013_9	11/02/2013	14/03/2013		470	300	x	24 ± 6	1517	47 ± 15	63 ± 45	x	3,5 ± 0,6	111
2013_11	11/02/2013	08/10/2013		556	256	x	23 ± 7	10151	44 ± 13	22 ± 32	x	3,2 ± 0,8	730
2013_12	17/02/2013	07/10/2013	10	1150	375	x	19 ± 7	7728	36 ± 12	31 ± 21	No	3,0 ± 0,9	657
2013_13	10/02/2013	20/04/2013		600	321	x	23 ± 6	3501	50 ± 17	50 ± 37	x	3,4 ± 0,8	220
2013_14	17/03/2013	24/11/2013	11	300	270	x	20 ± 8	10074	42 ± 16	19 ± 32	x	3,2 ± 0,8	660
2014_7	26/12/2013	23/10/2014	7 then South	405	277	x	19 ± 9	11722	46 ± 21	32 ± 32	x	3,4 ± 0,7	413
2014_9	29/12/2013	11/09/2014		700	322	x	12 ± 6	4233	22 ± 10	35 ± 32	x	2,6 ± 1	471
2014_10	27/12/2013	27/09/2014	6 then North	700	306	x	14 ± 8	7876	35 ± 14	27 ± 36	x	3, ± 0,9	676
Mean ± SD				559 ± 244	293 ± 39		18 ± 9		41 ± 19	32 ± 34		2,8 ± 1	

Sum						21		136599			18		8568

Table S3. Definition criteria for water masses as determined from CTD-SRDL data collected by 21 post-moult male SESs. Temperature, salinity and pressure data were analysed at the bottom phase of dives (see Methods). Data spans 2004 to 2014 along tracks from 55°S to the Antarctic continent and from 30 to 150°E.

Acronym	Type of water mass	Neutral density γn (kg.m-3)	Potential Temperature θ (°C)	Depth D (m)	Zone
AASW	Antarctic Surface Water	$\gamma n \leq 28.0$			
CDW	Circumpolar Deep Water	$28.0 < \gamma n < 28.27$	$\theta > 1.5$		
mCDW	modified Circumpolar Deep Water	$28.0 < \gamma n < 28.27$	$-1.8 < \theta \leq 1.5$		
mSW-north	modified Shelf Water (north of the shelf break)	$\gamma n \geq 28.27$		D < 2500 m	
mSW-south	modified Shelf Water (south of the shelf break)	$\gamma n \geq 28.27$	$\theta > -1.8$		Antarctic Shelf
AABW	Antarctic Bottom Water	$\gamma n \geq 28.27$		D ≥ 2500 m	
ISW	Ice Shelf Water		$\theta \leq -1.95$		
DSW	Dense Shelf Water	$\gamma n \geq 28.27$	$-1.95 < \theta \leq -1.8$		
LSSW	Low Salinity Shelf Water (south of the shelf break)	$28.0 < \gamma n < 28.27$	$-1.95 < \theta < -1.8$		Antarctic Shelf

Table S4. Summary of regression coefficients from the 6 linear mixed effect models (LMMs). Individuals were included as a random intercept. Coefficients are presented ± Standard Error with their p-value associated. Significant parameters ($p < 0.01$) are denoted by bold characters. All are factor variables, thus coefficients are given relative to the first level of factor which is “inside polynyas” for models 1, 2, 3, and “outside polynyas” for models 4, 5, 6. Models 1 to 6 were based on dive data on a total of 23 males, (taking one dive every three dive to allow model computation, see the Methods section for more information).

**Model 1 (n = 42217, n = 23): Hunting time ~ Position (inside or outside polynyas)**		
	**Coefficient ± SE**	**Coefficient *p***
intercept	38.3 ± 7.8	0
Outside	-50.2 ± 8.7	**0**
**Model 2 (n = 20793, n = 21): Maximal depth ~ Position (inside or outside polynyas on the Antarctic shelf)**		
	**Coefficient ± SE**	**Coefficient *p***
intercept	-35.4 ± 8.5	0
Outside	15.1 ± 4.1	$\mathbf{2.10^{-4}}$
**Model 3 (n = 20793, n = 21): Dive duration ~ Position (inside or outside polynyas on the Antarctic shelf)**		
	**Coefficient ± SE**	**Coefficient *p***
intercept	12 ± 8.9	0.18
Outside	-23 ± 11.6	0.0475
**Model 4 (n = 42217, n = 23): Hunting time ~ Position (outside polynyas or inside polynyas in cores 1, 2 or 3)**		
	**Coefficient ± SE**	**Coefficient *p***
intercept	-12.9 ± 4.8	0.0075
Core 1	27.6 ± 11	0.0122
Core 2	87.6 ± 12.4	**0**
Core 3	31.9 ± 54	0.2445
**Model 5 (n = 20793, n = 21): Maximal depth ~ Position (outside or inside polynyas in cores 1, 2 or 3 on the Antarctic shelf)**		
	**Coefficient ± SE**	**Coefficient *p***
intercept	-19.1 ± 8.3	0.0207
Core 1	-13.1 ± 4.5	**0.0035**
Core 2	-31.6 ± 5.6	**0**
Core 3	21.3 ± 9.9	0.0312
**Model 6 (n = 20793, n = 21): Dive duration ~ Position (outside or inside polynyas in cores 1, 2 or 3 on the Antarctic shelf)**		
	**Coefficient ± SE**	**Coefficient *p***
intercept	-11.6 ± 8.5	0.1747
Core 1	11.3 ± 14	0.4218
Core 2	35.8 ± 15	0.0167
Core 3	29.5 ± 30.9	0.3399

Table S5. Summary of the GAMM relating hunting time to the upper ocean stratification for three individuals visiting the Cape Poinsett, Mackenzie and West Ice Shelf polynya. Individuals were included as a random intercept and slope. Significant parameters are denoted by bold characters. The model was based on CTD casts associated with dive characteristics (see the Methods section for more information).

**Model 1 (n = 988, n = 3): Hunting time ~ s(log(stratification))**		
	**edf**	**Coefficient *p***
s(logstratif100)	5.1	**0.008**
s(seal,logstratif100)	7.7	**0.01**

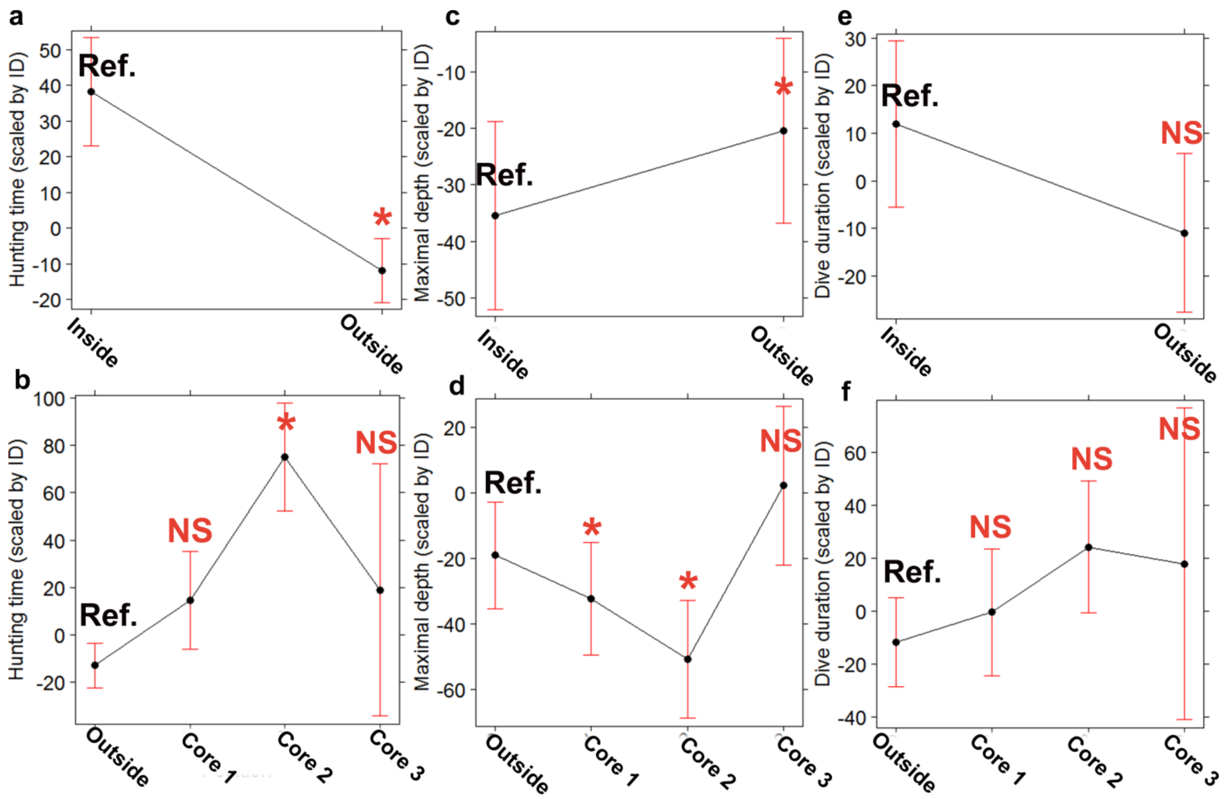

Fig. S1. Change in hunting times, maximal depth and dive durations inside and outside polynyas and within the different cores of the polynya. Fitted relationships from the LMMs relating hunting time to the seal position are represented: inside versus outside polynyas in panel (a), and outside and in cores 1, 2 and 3 of the polynyas in panel (b). LMMS (panels a, b) were computed based on dive data (19 and 23 SESs inside and outside polynyas respectively). Fitted relationships from the LMMs relating maximal diving depth and dive duration to seal position in the Antarctic shelf are also shown: inside versus outside polynyas in panels (c & e), and outside and cores 1, 2 and 3 of the polynyas in panels (d & f). LMMS (panels c, d, e & f) were computed based on dive data in the Antarctic shelf (19 and 21 SESs inside and outside polynyas respectively on the shelf). The red star indicates the significance of the factor relative to the reference factor (Ref.) for each plot. NS means not significantly different from the reference factor.

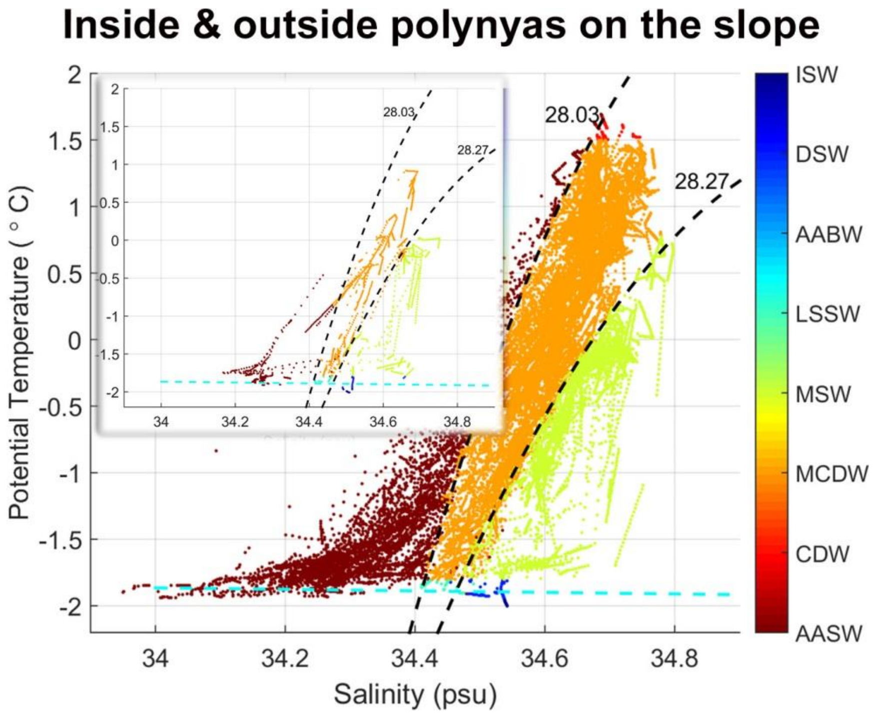

Fig. S2. Temperature salinity diagrams of hydrologic properties sampled by the 20 post-moult Kerguelen male SESs visiting the Antarctic slope from 2004 to 2014 CTD casts. All water masses sampled at the bottom phase of dives outside of polynyas on the slope region by 20 seals (CTD casts = 1389) are represented in the main temperature salinity diagram while the inset panel represents water masses sampled inside polynyas on the slope region by 6 seals (CTD casts = 118). Acronyms and definitions of water mass classes can be found in Supplementary, Table S3.

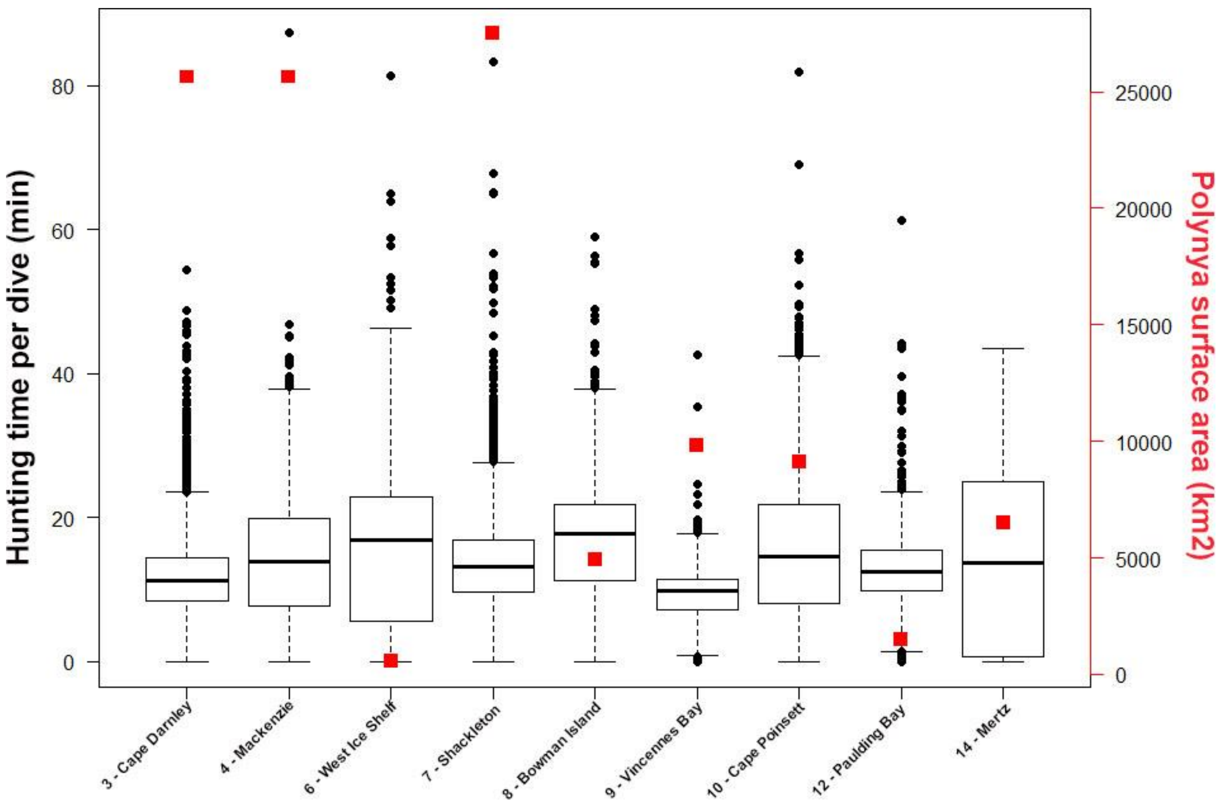

Fig. S3. Relation between polynya maximal surface extent area and SES hunting time per dive. Boxplots of hunting time per dive (expressed in minutes) are represented for each of the 9 polynyas visited by the 18 SESs from 2004 to 2014. The red squares indicate the maximal surface extent area (expressed in km^2) for each polynya based on the larger yearly sea ice production contour (2.5 $m.y^{-1}$).

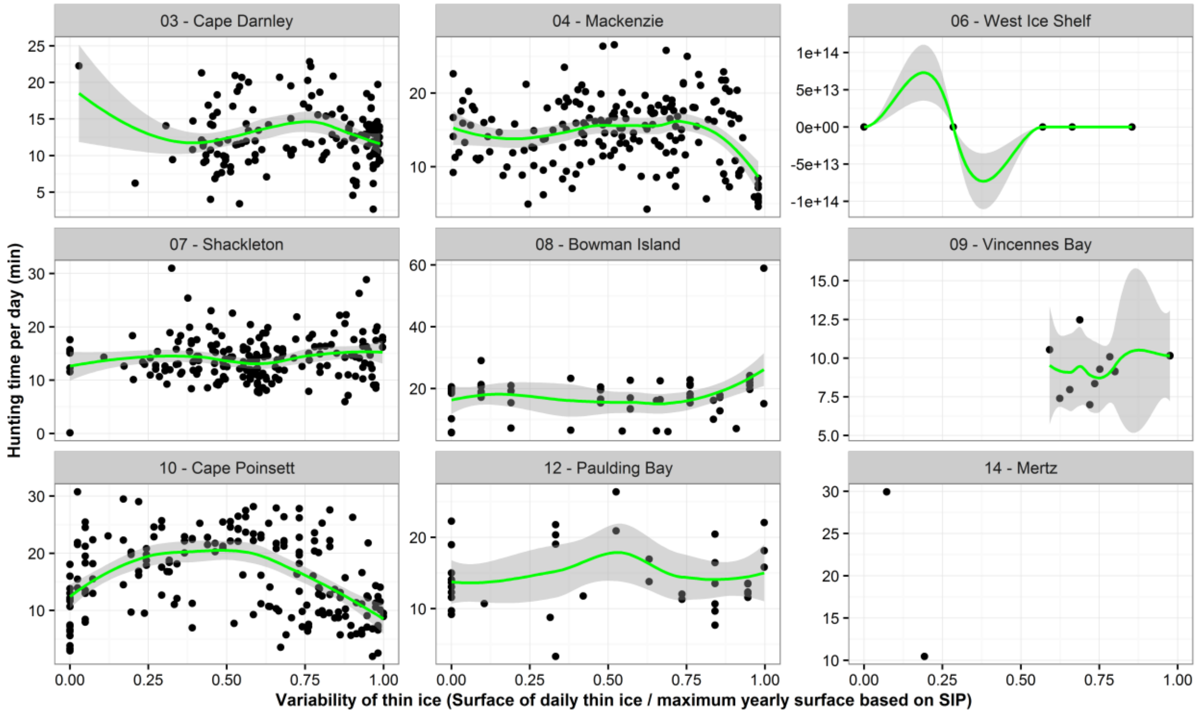

Fig. S4. Relation between the variability of polynya surface extent area at a daily scale and SES hunting time per dive averaged per day. Scatter plots representing hunting time per dive (expressed in minutes) averaged per day for each of the 9 polynyas visited by the 18 SESs from 2004 to 2014 in relation with the variability in surface extent area of polynya. The variability is expressed as thin ice variability, in a given polynya it represents the division of the daily thin ice surface extent area (defined by thin ice thickness above 0 and inferior to 0.2 m) by the maximal surface extent area for the given polynya (based on the larger yearly sea ice production contour (2.5 $m.y^{-1}$). The green line represents a smoothed conditional mean of the observations by fitting a polynomial regression using least squares with the package *ggplot2* and the argument *geom_smooth* from R Development Core Team. The grey bandwidth represents the 95% confidence level interval for predictions. Lack of observations for polynyas 6, 9 and 14 led to the absence or incorrect smoothed means.

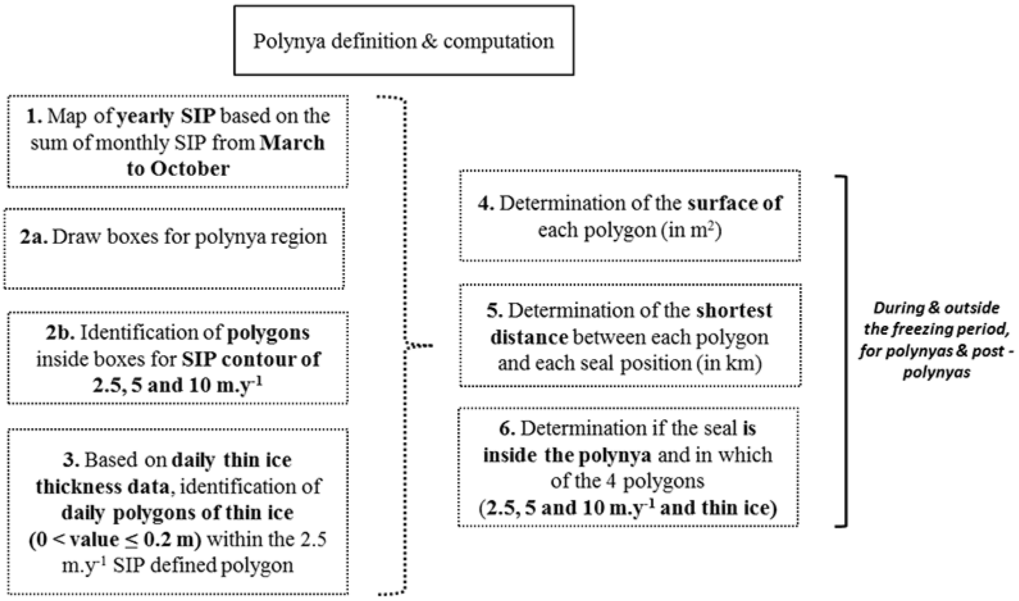

Fig. S5. Schematic representing the steps for defining polynyas and the calculation of SES polynya use. SIP = sea ice production ($m.y^{-1}$).
